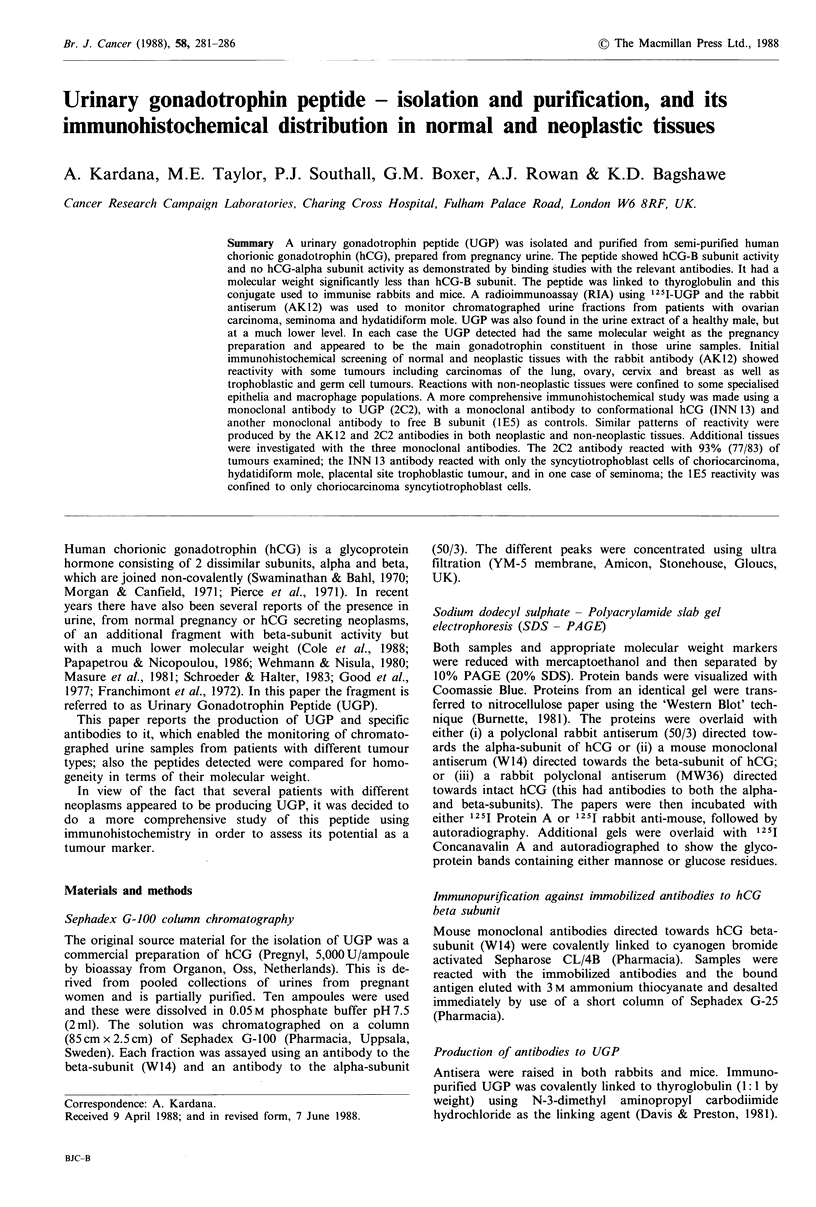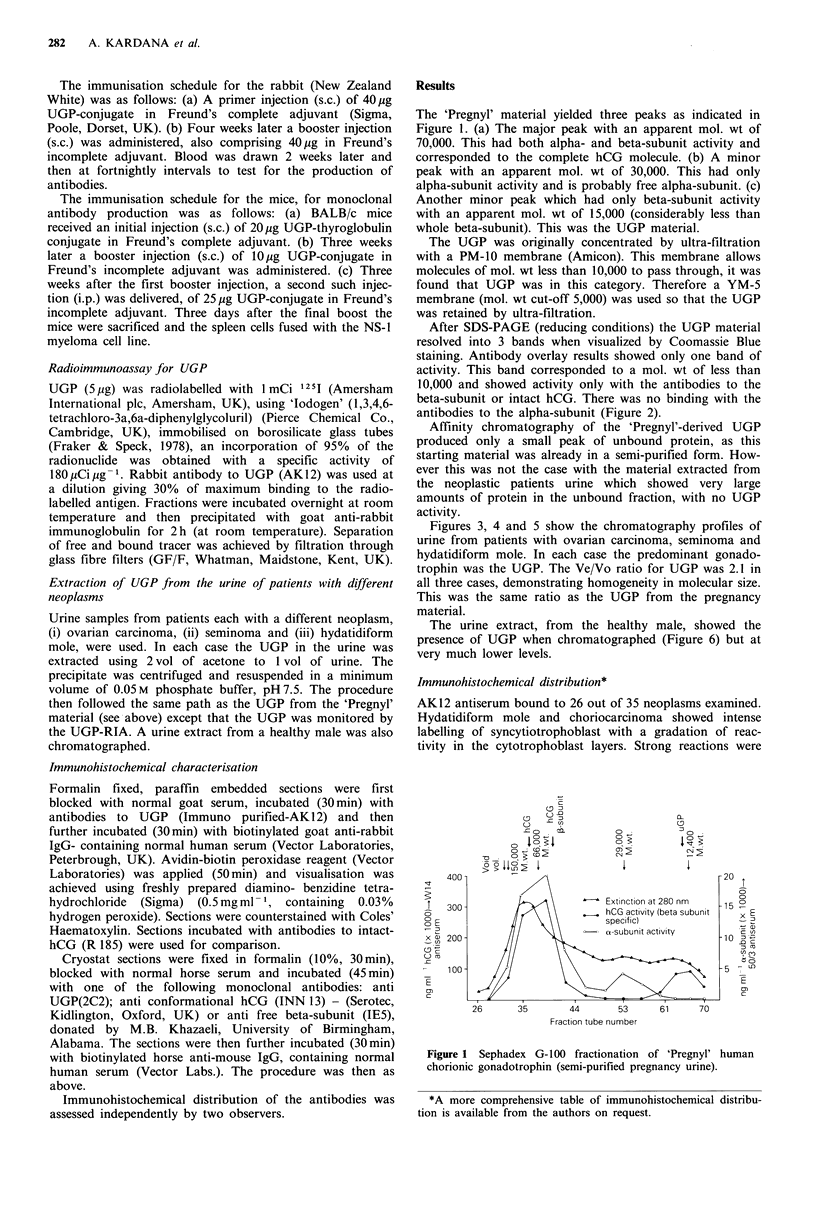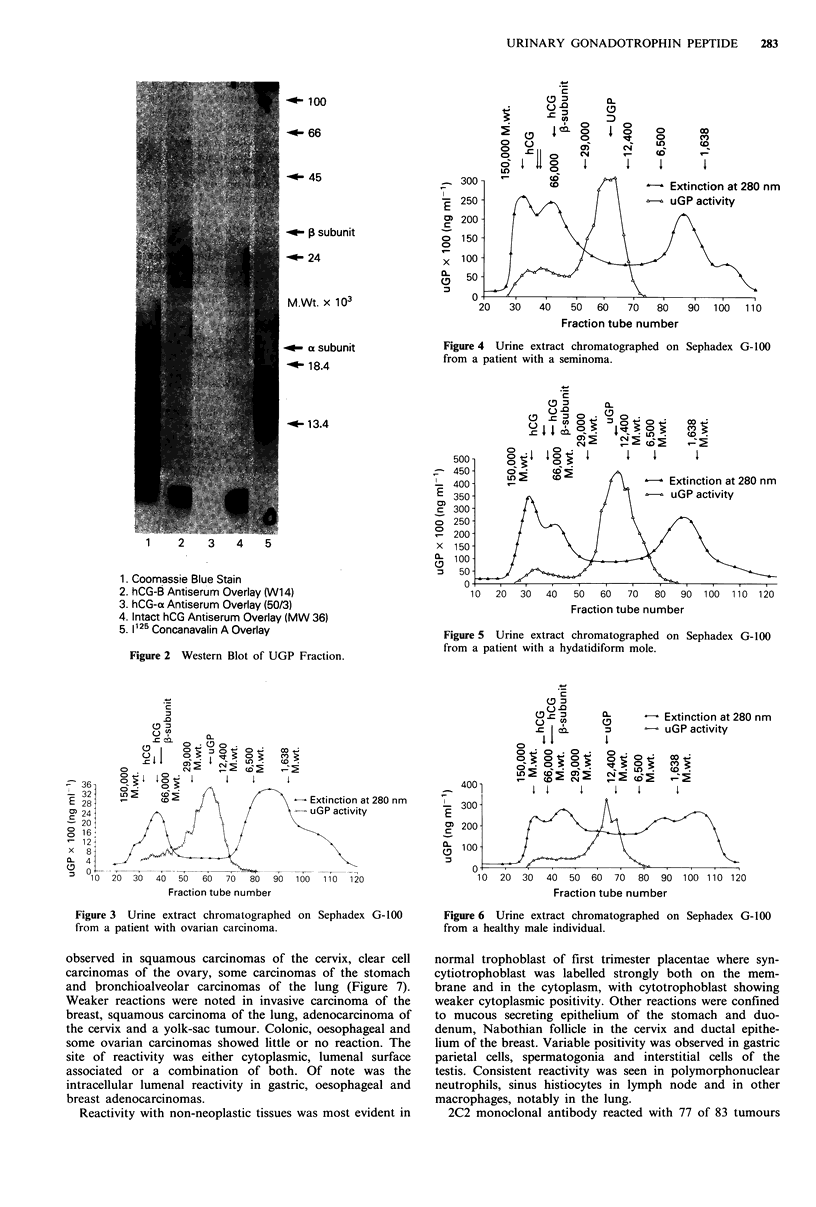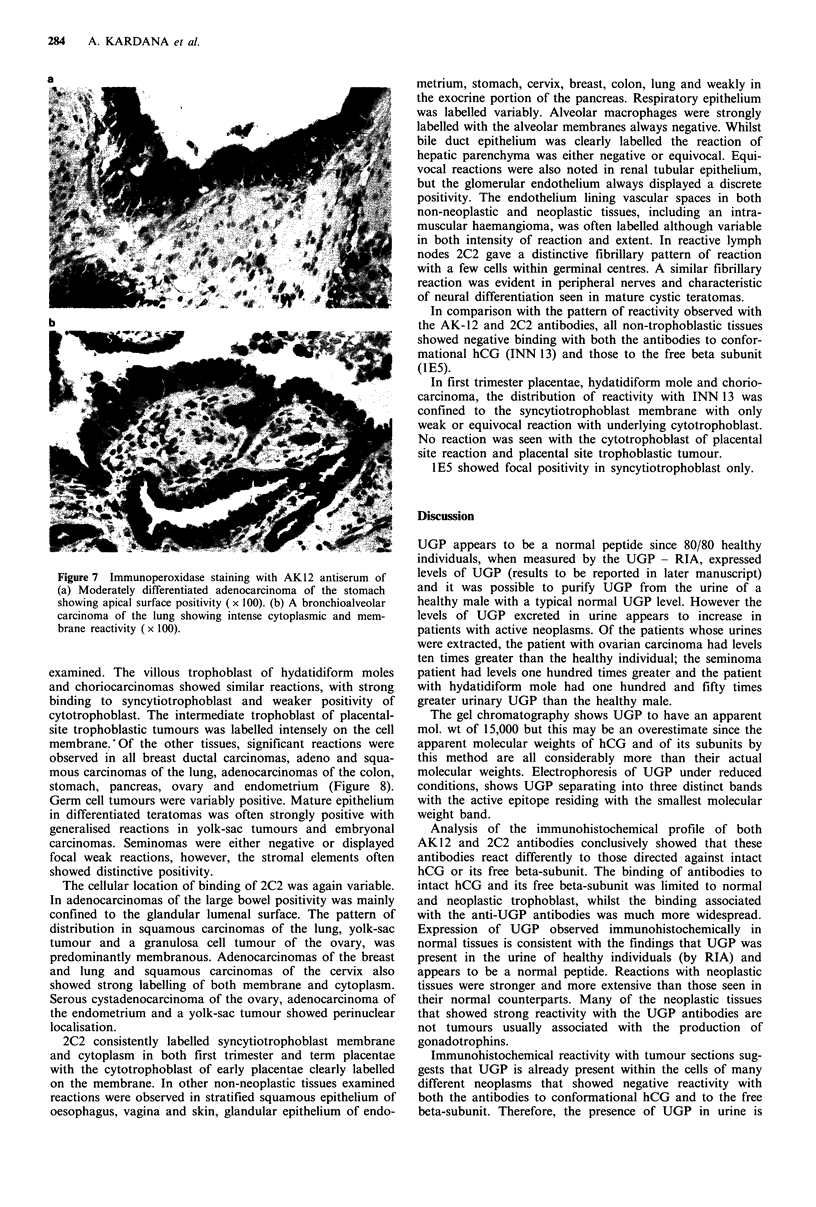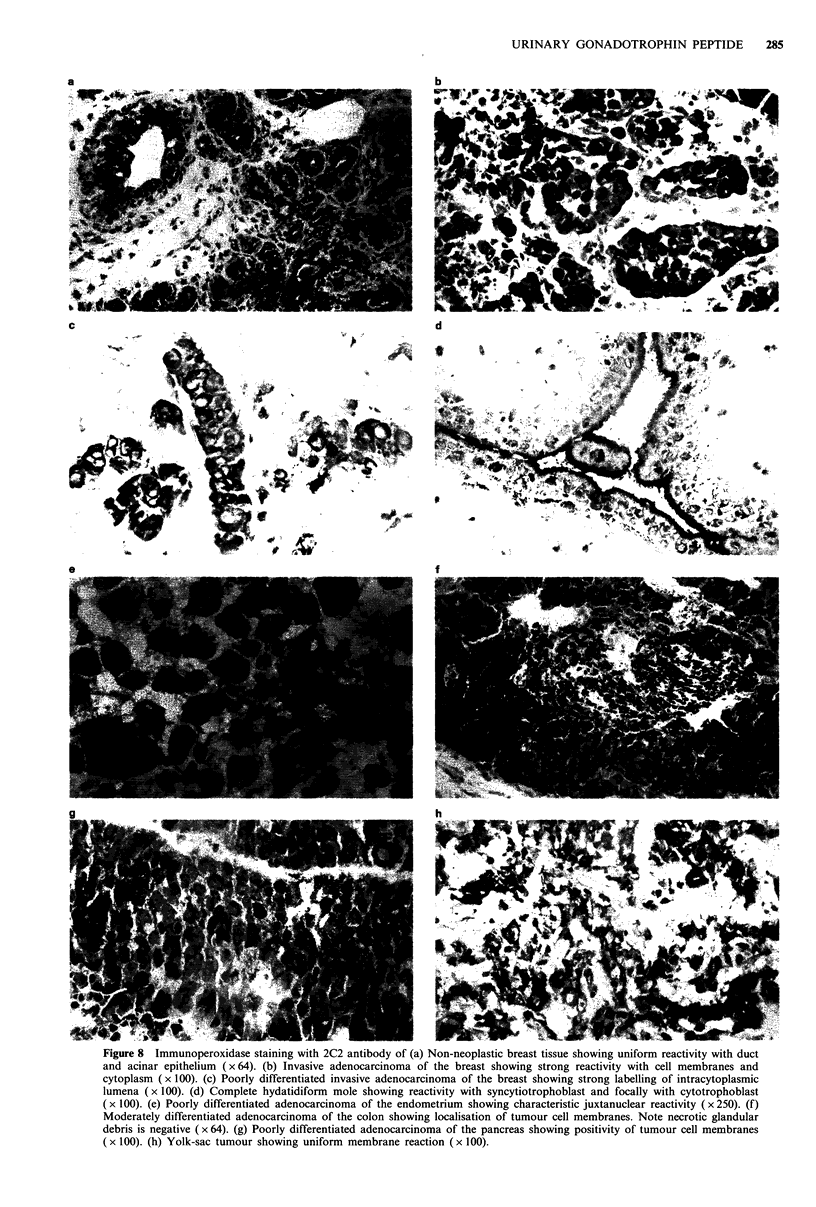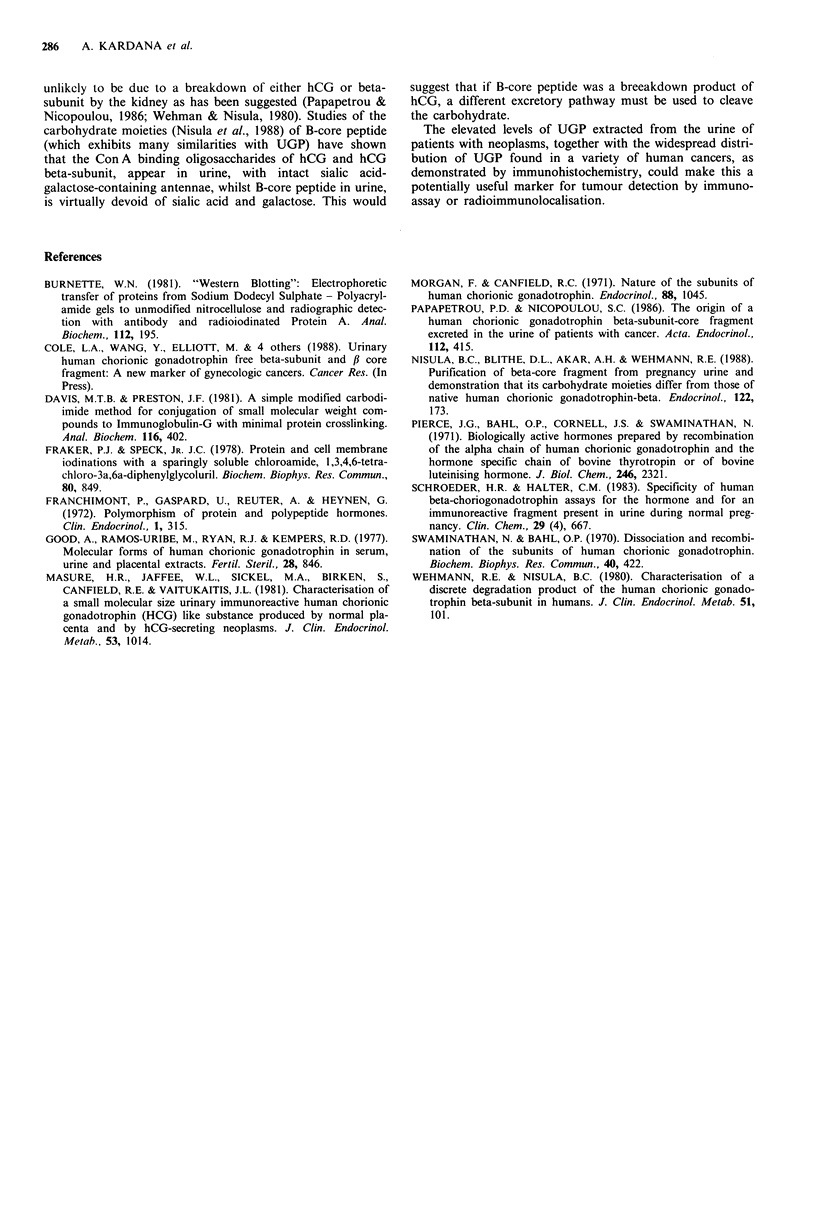# Urinary gonadotrophin peptide--isolation and purification, and its immunohistochemical distribution in normal and neoplastic tissues.

**DOI:** 10.1038/bjc.1988.204

**Published:** 1988-09

**Authors:** A. Kardana, M. E. Taylor, P. J. Southall, G. M. Boxer, A. J. Rowan, K. D. Bagshawe

**Affiliations:** Cancer Research Campaign Laboratories, Charing Cross Hospital, London, UK.

## Abstract

**Images:**


*Br. J. Cancer* (1988), **58**, 281–286 © The Macmillan Press Ltd., 1988

### Urinary gonadotrophin peptide – isolation and purification, and its immunohistochemical distribution in normal and neoplastic tissues

A. Kardana, M.E. Taylor, P.J. Southall, G.M. Boxer, A.J. Rowan & K.D. Bagshawe

*Cancer Research Campaign Laboratories, Charing Cross Hospital, Fulham Palace Road, London W6 8RF, UK.*

**Summary** A urinary gonadotrophin peptide (UGP) was isolated and purified from semi-purified human chorionic gonadotrophin (hCG), prepared from pregnancy urine. The peptide showed hCG-B subunit activity and no hCG-alpha subunit activity as demonstrated by binding studies with the relevant antibodies. It had a molecular weight significantly less than hCG-B subunit. The peptide was linked to thyroglobulin and this conjugate used to immunise rabbits and mice. A radioimmunoassay (RIA) using $^{125}$I-UGP and the rabbit antiserum (AK12) was used to monitor chromatographed urine fractions from patients with ovarian carcinoma, seminoma and hydatidiform mole. UGP was also found in the urine extract of a healthy male, but at a much lower level. In each case the UGP detected had the same molecular weight as the pregnancy preparation and appeared to be the main gonadotrophin constituent in those urine samples. Initial immunohistochemical screening of normal and neoplastic tissues with the rabbit antibody (AK12) showed reactivity with some tumours including carcinomas of the lung, ovary, cervix and breast as well as trophoblastic and germ cell tumours. Reactions with non-neoplastic tissues were confined to some specialised epithelia and macrophage populations. A more comprehensive immunohistochemical study was made using a monoclonal antibody to UGP (2C2), with a monoclonal antibody to conformational hCG (INN 13) and another monoclonal antibody to free B subunit (1E5) as controls. Similar patterns of reactivity were produced by the AK12 and 2C2 antibodies in both neoplastic and non-neoplastic tissues. Additional tissues were investigated with the three monoclonal antibodies. The 2C2 antibody reacted with 93% (77/83) of tumours examined; the INN 13 antibody reacted with only the syncytiotrophoblast cells of choriocarcinoma, hydatidiform mole, placental site trophoblastic tumour, and in one case of seminoma; the 1E5 reactivity was confined to only choriocarcinoma syncytiotrophoblast cells.

Human chorionic gonadotrophin (hCG) is a glycoprotein hormone consisting of 2 dissimilar subunits, alpha and beta, which are joined non-covalently (Swaminathan & Bahl, 1970; Morgan & Canfield, 1971; Pierce *et al.*, 1971). In recent years there have also been several reports of the presence in urine, from normal pregnancy or hCG secreting neoplasms, of an additional fragment with beta-subunit activity but with a much lower molecular weight (Cole *et al.*, 1988; Papapetrou & Nicopoulou, 1986; Wehmann & Nisula, 1980; Masure *et al.*, 1981; Schroeder & Halter, 1983; Good *et al.*, 1977; Franchimont *et al.*, 1972). In this paper the fragment is referred to as Urinary Gonadotrophin Peptide (UGP).

This paper reports the production of UGP and specific antibodies to it, which enabled the monitoring of chromatographed urine samples from patients with different tumour types; also the peptides detected were compared for homogeneity in terms of their molecular weight.

In view of the fact that several patients with different neoplasms appeared to be producing UGP, it was decided to do a more comprehensive study of this peptide using immunohistochemistry in order to assess its potential as a tumour marker.

### Materials and methods

### *Sephadex G-100 column chromatography*

The original source material for the isolation of UGP was a commercial preparation of hCG (Pregnyl, 5,000 U/ampoule by bioassay from Organon, Oss, Netherlands). This is derived from pooled collections of urines from pregnant women and is partially purified. Ten ampoules were used and these were dissolved in 0.05 M phosphate buffer pH 7.5 (2 ml). The solution was chromatographed on a column (85 cm × 2.5 cm) of Sephadex G-100 (Pharmacia, Uppsala, Sweden). Each fraction was assayed using an antibody to the beta-subunit (W14) and an antibody to the alpha-subunit (50/3). The different peaks were concentrated using ultra filtration (YM-5 membrane, Amicon, Stonehouse, Gloucs, UK).

### *Sodium dodecyl sulphate – Polyacrylamide slab gel electrophoresis (SDS – PAGE)*

Both samples and appropriate molecular weight markers were reduced with mercaptoethanol and then separated by 10% PAGE (20% SDS). Protein bands were visualized with Coomassie Blue. Proteins from an identical gel were transferred to nitrocellulose paper using the 'Western Blot' technique (Burnette, 1981). The proteins were overlaid with either (i) a polyclonal rabbit antiserum (50/3) directed towards the alpha-subunit of hCG or (ii) a mouse monoclonal antiserum (W14) directed towards the beta-subunit of hCG; or (iii) a rabbit polyclonal antiserum (MW36) directed towards intact hCG (this had antibodies to both the alpha- and beta-subunits). The papers were then incubated with either $^{125}$I Protein A or $^{125}$I rabbit anti-mouse, followed by autoradiography. Additional gels were overlaid with $^{125}$I Concanavalin A and autoradiographed to show the glycoprotein bands containing either mannose or glucose residues.

### *Immunopurification against immobilized antibodies to hCG beta subunit*

Mouse monoclonal antibodies directed towards hCG beta-subunit (W14) were covalently linked to cyanogen bromide activated Sepharose CL/4B (Pharmacia). Samples were reacted with the immobilized antibodies and the bound antigen eluted with 3 M ammonium thiocyanate and desalted immediately by use of a short column of Sephadex G-25 (Pharmacia).

### *Production of antibodies to UGP*

Antisera were raised in both rabbits and mice. Immunopurified UGP was covalently linked to thyroglobulin (1:1 by weight) using N-3-dimethyl aminopropyl carbodiimide hydrochloride as the linking agent (Davis & Preston, 1981).

Correspondence: A. Kardana.
Received 9 April 1988; and in revised form, 7 June 1988.

The immunisation schedule for the rabbit (New Zealand White) was as follows: (a) A primer injection (s.c.) of 40 μg UGP-conjugate in Freund's complete adjuvant (Sigma, Poole, Dorset, UK). (b) Four weeks later a booster injection (s.c.) was administered, also comprising 40 μg in Freund's incomplete adjuvant. Blood was drawn 2 weeks later and then at fortnightly intervals to test for the production of antibodies.

The immunisation schedule for the mice, for monoclonal antibody production was as follows: (a) BALB/c mice received an initial injection (s.c.) of 20 μg UGP-thyroglobulin conjugate in Freund's complete adjuvant. (b) Three weeks later a booster injection (s.c.) of 10 μg UGP-conjugate in Freund's incomplete adjuvant was administered. (c) Three weeks after the first booster injection, a second such injection (i.p.) was delivered, of 25 μg UGP-conjugate in Freund's incomplete adjuvant. Three days after the final boost the mice were sacrificed and the spleen cells fused with the NS-1 myeloma cell line.

*Radioimmunoassay for UGP*

UGP (5 μg) was radiolabelled with 1 mCi $^{125}I$ (Amersham International plc, Amersham, UK), using 'Iodogen' (1,3,4,6-tetrachloro-3a,6a-diphenylglycoluril) (Pierce Chemical Co., Cambridge, UK), immobilised on borosilicate glass tubes (Fraker & Speck, 1978), an incorporation of 95% of the radionuclide was obtained with a specific activity of 180 $\mu Ci\ \mu g^{-1}$. Rabbit antibody to UGP (AK12) was used at a dilution giving 30% of maximum binding to the radiolabelled antigen. Fractions were incubated overnight at room temperature and then precipitated with goat anti-rabbit immunoglobulin for 2 h (at room temperature). Separation of free and bound tracer was achieved by filtration through glass fibre filters (GF/F, Whatman, Maidstone, Kent, UK).

*Extraction of UGP from the urine of patients with different neoplasms*

Urine samples from patients each with a different neoplasm, (i) ovarian carcinoma, (ii) seminoma and (iii) hydatidiform mole, were used. In each case the UGP in the urine was extracted using 2 vol of acetone to 1 vol of urine. The precipitate was centrifuged and resuspended in a minimum volume of 0.05 M phosphate buffer, pH 7.5. The procedure then followed the same path as the UGP from the 'Pregnyl' material (see above) except that the UGP was monitored by the UGP-RIA. A urine extract from a healthy male was also chromatographed.

*Immunohistochemical characterisation*

Formalin fixed, paraffin embedded sections were first blocked with normal goat serum, incubated (30 min) with antibodies to UGP (Immuno purified-AK12) and then further incubated (30 min) with biotinylated goat anti-rabbit IgG- containing normal human serum (Vector Laboratories, Peterbrough, UK). Avidin-biotin peroxidase reagent (Vector Laboratories) was applied (50 min) and visualisation was achieved using freshly prepared diamino- benzidine tetrahydrochloride (Sigma) (0.5 $mg\ ml^{-1}$, containing 0.03% hydrogen peroxide). Sections were counterstained with Coles' Haematoxylin. Sections incubated with antibodies to intact-hCG (R 185) were used for comparison.

Cryostat sections were fixed in formalin (10%, 30 min), blocked with normal horse serum and incubated (45 min) with one of the following monoclonal antibodies: anti UGP(2C2); anti conformational hCG (INN 13) – (Serotec, Kidlington, Oxford, UK) or anti free beta-subunit (IE5), donated by M.B. Khazaeli, University of Birmingham, Alabama. The sections were then further incubated (30 min) with biotinylated horse anti-mouse IgG, containing normal human serum (Vector Labs.). The procedure was then as above.

Immunohistochemical distribution of the antibodies was assessed independently by two observers.

**Results**

The 'Pregnyl' material yielded three peaks as indicated in Figure 1. (a) The major peak with an apparent mol. wt of 70,000. This had both alpha- and beta-subunit activity and corresponded to the complete hCG molecule. (b) A minor peak with an apparent mol. wt of 30,000. This had only alpha-subunit activity and is probably free alpha-subunit. (c) Another minor peak which had only beta-subunit activity with an apparent mol. wt of 15,000 (considerably less than whole beta-subunit). This was the UGP material.

The UGP was originally concentrated by ultra-filtration with a PM-10 membrane (Amicon). This membrane allows molecules of mol. wt less than 10,000 to pass through, it was found that UGP was in this category. Therefore a YM-5 membrane (mol. wt cut-off 5,000) was used so that the UGP was retained by ultra-filtration.

After SDS-PAGE (reducing conditions) the UGP material resolved into 3 bands when visualized by Coomassie Blue staining. Antibody overlay results showed only one band of activity. This band corresponded to a mol. wt of less than 10,000 and showed activity only with the antibodies to the beta-subunit or intact hCG. There was no binding with the antibodies to the alpha-subunit (Figure 2).

Affinity chromatography of the 'Pregnyl'-derived UGP produced only a small peak of unbound protein, as this starting material was already in a semi-purified form. However this was not the case with the material extracted from the neoplastic patients urine which showed very large amounts of protein in the unbound fraction, with no UGP activity.

Figures 3, 4 and 5 show the chromatography profiles of urine from patients with ovarian carcinoma, seminoma and hydatidiform mole. In each case the predominant gonadotrophin was the UGP. The Ve/Vo ratio for UGP was 2.1 in all three cases, demonstrating homogeneity in molecular size. This was the same ratio as the UGP from the pregnancy material.

The urine extract, from the healthy male, showed the presence of UGP when chromatographed (Figure 6) but at very much lower levels.

*Immunohistochemical distribution**

AK12 antiserum bound to 26 out of 35 neoplasms examined. Hydatidiform mole and choriocarcinoma showed intense labelling of syncytiotrophoblast with a gradation of reactivity in the cytotrophoblast layers. Strong reactions were

**Figure 1** Sephadex G-100 fractionation of 'Pregnyl' human chorionic gonadotrophin (semi-purified pregnancy urine).

*A more comprehensive table of immunohistochemical distribution is available from the authors on request.

**Figure 2** Western Blot of UGP Fraction.

**Figure 3** Urine extract chromatographed on Sephadex G-100 from a patient with ovarian carcinoma.

**Figure 4** Urine extract chromatographed on Sephadex G-100 from a patient with a seminoma.

**Figure 5** Urine extract chromatographed on Sephadex G-100 from a patient with a hydatidiform mole.

**Figure 6** Urine extract chromatographed on Sephadex G-100 from a healthy male individual.

observed in squamous carcinomas of the cervix, clear cell carcinomas of the ovary, some carcinomas of the stomach and bronchioalveolar carcinomas of the lung (Figure 7). Weaker reactions were noted in invasive carcinoma of the breast, squamous carcinoma of the lung, adenocarcinoma of the cervix and a yolk-sac tumour. Colonic, oesophageal and some ovarian carcinomas showed little or no reaction. The site of reactivity was either cytoplasmic, lumenal surface associated or a combination of both. Of note was the intracellular lumenal reactivity in gastric, oesophageal and breast adenocarcinomas.

Reactivity with non-neoplastic tissues was most evident in normal trophoblast of first trimester placentae where syncytiotrophoblast was labelled strongly both on the membrane and in the cytoplasm, with cytotrophoblast showing weaker cytoplasmic positivity. Other reactions were confined to mucous secreting epithelium of the stomach and duodenum, Nabothian follicle in the cervix and ductal epithelium of the breast. Variable positivity was observed in gastric parietal cells, spermatogonia and interstitial cells of the testis. Consistent reactivity was seen in polymorphonuclear neutrophils, sinus histiocytes in lymph node and in other macrophages, notably in the lung.

2C2 monoclonal antibody reacted with 77 of 83 tumours

**Figure 7** Immunoperoxidase staining with AK12 antiserum of (a) Moderately differentiated adenocarcinoma of the stomach showing apical surface positivity ( × 100). (b) A bronchioalveolar carcinoma of the lung showing intense cytoplasmic and membrane reactivity ( × 100).

examined. The villous trophoblast of hydatidiform moles and choriocarcinomas showed similar reactions, with strong binding to syncytiotrophoblast and weaker positivity of cytotrophoblast. The intermediate trophoblast of placental-site trophoblastic tumours was labelled intensely on the cell membrane. Of the other tissues, significant reactions were observed in all breast ductal carcinomas, adeno and squamous carcinomas of the lung, adenocarcinomas of the colon, stomach, pancreas, ovary and endometrium (Figure 8). Germ cell tumours were variably positive. Mature epithelium in differentiated teratomas was often strongly positive with generalised reactions in yolk-sac tumours and embryonal carcinomas. Seminomas were either negative or displayed focal weak reactions, however, the stromal elements often showed distinctive positivity.

The cellular location of binding of 2C2 was again variable. In adenocarcinomas of the large bowel positivity was mainly confined to the glandular lumenal surface. The pattern of distribution in squamous carcinomas of the lung, yolk-sac tumour and a granulosa cell tumour of the ovary, was predominantly membranous. Adenocarcinomas of the breast and lung and squamous carcinomas of the cervix also showed strong labelling of both membrane and cytoplasm. Serous cystadenocarcinoma of the ovary, adenocarcinoma of the endometrium and a yolk-sac tumour showed perinuclear localisation.

2C2 consistently labelled syncytiotrophoblast membrane and cytoplasm in both first trimester and term placentae with the cytotrophoblast of early placentae clearly labelled on the membrane. In other non-neoplastic tissues examined reactions were observed in stratified squamous epithelium of oesophagus, vagina and skin, glandular epithelium of endometrium, stomach, cervix, breast, colon, lung and weakly in the exocrine portion of the pancreas. Respiratory epithelium was labelled variably. Alveolar macrophages were strongly labelled with the alveolar membranes always negative. Whilst bile duct epithelium was clearly labelled the reaction of hepatic parenchyma was either negative or equivocal. Equivocal reactions were also noted in renal tubular epithelium, but the glomerular endothelium always displayed a discrete positivity. The endothelium lining vascular spaces in both non-neoplastic and neoplastic tissues, including an intramuscular haemangioma, was often labelled although variable in both intensity of reaction and extent. In reactive lymph nodes 2C2 gave a distinctive fibrillary pattern of reaction with a few cells within germinal centres. A similar fibrillary reaction was evident in peripheral nerves and characteristic of neural differentiation seen in mature cystic teratomas.

In comparison with the pattern of reactivity observed with the AK-12 and 2C2 antibodies, all non-trophoblastic tissues showed negative binding with both the antibodies to conformational hCG (INN 13) and those to the free beta subunit (1E5).

In first trimester placentae, hydatidiform mole and choriocarcinoma, the distribution of reactivity with INN 13 was confined to the syncytiotrophoblast membrane with only weak or equivocal reaction with underlying cytotrophoblast. No reaction was seen with the cytotrophoblast of placental site reaction and placental site trophoblastic tumour.

1E5 showed focal positivity in syncytiotrophoblast only.

### Discussion

UGP appears to be a normal peptide since 80/80 healthy individuals, when measured by the UGP – RIA, expressed levels of UGP (results to be reported in later manuscript) and it was possible to purify UGP from the urine of a healthy male with a typical normal UGP level. However the levels of UGP excreted in urine appears to increase in patients with active neoplasms. Of the patients whose urines were extracted, the patient with ovarian carcinoma had levels ten times greater than the healthy individual; the seminoma patient had levels one hundred times greater and the patient with hydatidiform mole had one hundred and fifty times greater urinary UGP than the healthy male.

The gel chromatography shows UGP to have an apparent mol. wt of 15,000 but this may be an overestimate since the apparent molecular weights of hCG and of its subunits by this method are all considerably more than their actual molecular weights. Electrophoresis of UGP under reduced conditions, shows UGP separating into three distinct bands with the active epitope residing with the smallest molecular weight band.

Analysis of the immunohistochemical profile of both AK12 and 2C2 antibodies conclusively showed that these antibodies react differently to those directed against intact hCG or its free beta-subunit. The binding of antibodies to intact hCG and its free beta-subunit was limited to normal and neoplastic trophoblast, whilst the binding associated with the anti-UGP antibodies was much more widespread. Expression of UGP observed immunohistochemically in normal tissues is consistent with the findings that UGP was present in the urine of healthy individuals (by RIA) and appears to be a normal peptide. Reactions with neoplastic tissues were stronger and more extensive than those seen in their normal counterparts. Many of the neoplastic tissues that showed strong reactivity with the UGP antibodies are not tumours usually associated with the production of gonadotrophins.

Immunohistochemical reactivity with tumour sections suggests that UGP is already present within the cells of many different neoplasms that showed negative reactivity with both the antibodies to conformational hCG and to the free beta-subunit. Therefore, the presence of UGP in urine is

**Figure 8** Immunoperoxidase staining with 2C2 antibody of (a) Non-neoplastic breast tissue showing uniform reactivity with duct and acinar epithelium (×64). (b) Invasive adenocarcinoma of the breast showing strong reactivity with cell membranes and cytoplasm (×100). (c) Poorly differentiated invasive adenocarcinoma of the breast showing strong labelling of intracytoplasmic lumena (×100). (d) Complete hydatidiform mole showing reactivity with syncytiotrophoblast and focally with cytotrophoblast (×100). (e) Poorly differentiated adenocarcinoma of the endometrium showing characteristic juxtanuclear reactivity (×250). (f) Moderately differentiated adenocarcinoma of the colon showing localisation of tumour cell membranes. Note necrotic glandular debris is negative (×64). (g) Poorly differentiated adenocarcinoma of the pancreas showing positivity of tumour cell membranes (×100). (h) Yolk-sac tumour showing uniform membrane reaction (×100).

unlikely to be due to a breakdown of either hCG or beta-subunit by the kidney as has been suggested (Papapetrou & Nicopoulou, 1986; Wehman & Nisula, 1980). Studies of the carbohydrate moieties (Nisula *et al.*, 1988) of B-core peptide (which exhibits many similarities with UGP) have shown that the Con A binding oligosaccharides of hCG and hCG beta-subunit, appear in urine, with intact sialic acid-galactose-containing antennae, whilst B-core peptide in urine, is virtually devoid of sialic acid and galactose. This would suggest that if B-core peptide was a breeakdown product of hCG, a different excretory pathway must be used to cleave the carbohydrate.

The elevated levels of UGP extracted from the urine of patients with neoplasms, together with the widespread distribution of UGP found in a variety of human cancers, as demonstrated by immunohistochemistry, could make this a potentially useful marker for tumour detection by immunoassay or radioimmunolocalisation.

### References

BURNETTE, W.N. (1981). "Western Blotting": Electrophoretic transfer of proteins from Sodium Dodecyl Sulphate – Polyacrylamide gels to unmodified nitrocellulose and radiographic detection with antibody and radioiodinated Protein A. *Anal. Biochem.*, **112,** 195.

COLE, L.A., WANG, Y., ELLIOTT, M. & 4 others (1988). Urinary human chorionic gonadotrophin free beta-subunit and $\beta$ core fragment: A new marker of gynecologic cancers. *Cancer Res.* (In Press).

DAVIS, M.T.B. & PRESTON, J.F. (1981). A simple modified carbodiimide method for conjugation of small molecular weight compounds to Immunoglobulin-G with minimal protein crosslinking. *Anal. Biochem.* **116,** 402.

FRAKER, P.J. & SPECK, JR. J.C. (1978). Protein and cell membrane iodinations with a sparingly soluble chloroamide, 1,3,4,6-tetrachloro-3a,6a-diphenylglycoluril. *Biochem. Biophys. Res. Commun.*, **80,** 849.

FRANCHIMONT, P., GASPARD, U., REUTER, A. & HEYNEN, G. (1972). Polymorphism of protein and polypeptide hormones. *Clin. Endocrinol.*, **1,** 315.

GOOD, A., RAMOS-URIBE, M., RYAN, R.J. & KEMPERS, R.D. (1977). Molecular forms of human chorionic gonadotrophin in serum, urine and placental extracts. *Fertil. Steril.*, **28,** 846.

MASURE, H.R., JAFFEE, W.L., SICKEL, M.A., BIRKEN, S., CANFIELD, R.E. & VAITUKAITIS, J.L. (1981). Characterisation of a small molecular size urinary immunoreactive human chorionic gonadotrophin (HCG) like substance produced by normal placenta and by hCG-secreting neoplasms. *J. Clin. Endocrinol. Metab.*, **53,** 1014.

MORGAN, F. & CANFIELD, R.C. (1971). Nature of the subunits of human chorionic gonadotrophin. *Endocrinol.*, **88,** 1045.

PAPAPETROU, P.D. & NICOPOULOU, S.C. (1986). The origin of a human chorionic gonadotrophin beta-subunit-core fragment excreted in the urine of patients with cancer. *Acta. Endocrinol.*, **112,** 415.

NISULA, B.C., BLITHE, D.L., AKAR, A.H. & WEHMANN, R.E. (1988). Purification of beta-core fragment from pregnancy urine and demonstration that its carbohydrate moieties differ from those of native human chorionic gonadotrophin-beta. *Endocrinol.*, **122,** 173.

PIERCE, J.G., BAHL, O.P., CORNELL, J.S. & SWAMINATHAN, N. (1971). Biologically active hormones prepared by recombination of the alpha chain of human chorionic gonadotrophin and the hormone specific chain of bovine thyrotropin or of bovine luteinising hormone. *J. Biol. Chem.*, **246,** 2321.

SCHROEDER, H.R. & HALTER, C.M. (1983). Specificity of human beta-choriogonadotrophin assays for the hormone and for an immunoreactive fragment present in urine during normal pregnancy. *Clin. Chem.*, **29** (4), 667.

SWAMINATHAN, N. & BAHL, O.P. (1970). Dissociation and recombination of the subunits of human chorionic gonadotrophin. *Biochem. Biophys. Res. Commun.*, **40,** 422.

WEHMANN, R.E. & NISULA, B.C. (1980). Characterisation of a discrete degradation product of the human chorionic gonadotrophin beta-subunit in humans. *J. Clin. Endocrinol. Metab.* **51,** 101.